# Supplementary material for: Mechanisms governing the pioneering and redistribution capabilities of the non-classical pioneer PU.1
Source: Nat Commun. 2020 Jan 21;11:402. doi: 10.1038/s41467-019-13960-2 (PMC6972792; doi:10.1038/s41467-019-13960-2)
Supplement: Supplementary file 7 — Source data [file 41467_2019_13960_MOESM7_ESM.zip › Source_Data/Figure6/Figure6B_MotifScanOutput/FLI1_mutPU.1/homerResults.html]

/misc/data/analysis/project\_PU1/CTV1/motifs/basic/peaksFLI1\_DSG\_PU1mut\_merged/final - Homer de novo Motif Results


# Homer *de novo* Motif Results (/misc/data/analysis/project\_PU1/CTV1/motifs/basic/peaksFLI1\_DSG\_PU1mut\_merged/final)

Known Motif Enrichment Results  
Gene Ontology Enrichment Results  
If Homer is having trouble matching a motif to a known motif, try copy/pasting the matrix file into
STAMP  
More information on motif finding results: HOMER
| Description of Results
| Tips
  
Total target sequences = 10260  
Total background sequences = 38123  
\* - possible false positive  

|  |  |  |  |  |  |  |  |  |
| --- | --- | --- | --- | --- | --- | --- | --- | --- |
| Rank | Motif | P-value | log P-pvalue | % of Targets | % of Background | STD(Bg STD) | Best Match/Details | Motif File |
| 1 | C T G A T A G C T G A C C T A G T A C G C T G A C G T A T C A G A G C T T A C G T C A G | 1e-3291 | -7.578e+03 | 85.43% | 20.24% | 40.8bp (65.4bp) | Fli1(ETS)/CD8-FLI-ChIP-Seq(GSE20898)/Homer(0.978) More Information \| Similar Motifs Found | motif file (matrix) |
| 2 | T G A C A C T G A C T G C T G A G C T A C T A G A G C T C T A G | 1e-932 | -2.147e+03 | 30.71% | 5.57% | 51.0bp (65.0bp) | ETS1(ETS)/Jurkat-ETS1-ChIP-Seq(GSE17954)/Homer(0.858) More Information \| Similar Motifs Found | motif file (matrix) |
| 3 | G A C T C G A T C A T G G A C T T A C G A C T G A G C T A G C T C G A T | 1e-899 | -2.072e+03 | 44.39% | 13.73% | 48.9bp (62.2bp) | RUNX1(Runt)/Jurkat-RUNX1-ChIP-Seq(GSE29180)/Homer(0.989) More Information \| Similar Motifs Found | motif file (matrix) |
| 4 | G A T C C A T G C A T G T C G A A C G T A C T G G C T A A T C G C G A T T A G C C G T A A G T C | 1e-218 | -5.036e+02 | 10.16% | 2.44% | 52.2bp (58.0bp) | AP-1(bZIP)/ThioMac-PU.1-ChIP-Seq(GSE21512)/Homer(0.983) More Information \| Similar Motifs Found | motif file (matrix) |
| 5 | A C G T A C T G A C G T A C T G A C T G A T C G G T C A | 1e-208 | -4.790e+02 | 42.00% | 26.01% | 53.5bp (61.8bp) | MET28/MA0332.1/Jaspar(0.839) More Information \| Similar Motifs Found | motif file (matrix) |
| 6 | A C G T A T G C A C G T A C G T C G T A A C G T A G T C C G A T T A G C G C A T | 1e-190 | -4.387e+02 | 17.74% | 7.41% | 53.7bp (63.2bp) | Gata4(Zf)/Heart-Gata4-ChIP-Seq(GSE35151)/Homer(0.984) More Information \| Similar Motifs Found | motif file (matrix) |
| 7 | A C T G A C T G A G C T G A C T G A C T A G T C G T A C A T C G C A G T A C G T A C G T | 1e-158 | -3.642e+02 | 9.76% | 3.02% | 54.5bp (67.5bp) | PB0033.1\_Irf3\_1/Jaspar(0.870) More Information \| Similar Motifs Found | motif file (matrix) |
| 8 | T C A G G C A T C T A G A C T G C T A G A C T G A C T G G T C A C T A G C A T G | 1e-135 | -3.130e+02 | 24.78% | 14.12% | 54.4bp (61.4bp) | EGR1/MA0162.2/Jaspar(0.884) More Information \| Similar Motifs Found | motif file (matrix) |
| 9 | A G T C G C A T A G C T G A C T T A C G G C T A C G A T A T C G C A G T A C T G T A C G | 1e-122 | -2.823e+02 | 9.82% | 3.65% | 52.3bp (63.4bp) | Tcf4(HMG)/Hct116-Tcf4-ChIP-Seq(SRA012054)/Homer(0.910) More Information \| Similar Motifs Found | motif file (matrix) |
| 10 | A G T C C T G A A G T C C G T A C G A T A G T C A G T C A C T G A C T G G A C T A G C T C A G T A T C G G C A T | 1e-120 | -2.782e+02 | 5.05% | 1.06% | 46.0bp (61.5bp) | SPDEF/MA0686.1/Jaspar(0.835) More Information \| Similar Motifs Found | motif file (matrix) |
| 11 | C T G A T A G C G C T A C A T G A T C G T G C A C G A T C A T G G A C T A C G T C A G T | 1e-92 | -2.137e+02 | 3.94% | 0.84% | 57.6bp (60.4bp) | Ets1-distal(ETS)/CD4+-PolII-ChIP-Seq(Barski\_et\_al.)/Homer(0.782) More Information \| Similar Motifs Found | motif file (matrix) |
| 12 | C G T A T G C A T G A C A T G C C T A G C A G T G T C A | 1e-90 | -2.094e+02 | 7.51% | 2.83% | 56.7bp (62.3bp) | MF0009.1\_TRP(MYB)\_class/Jaspar(0.824) More Information \| Similar Motifs Found | motif file (matrix) |
| 13 | G A T C T G C A C A T G T G A C A G C T T C A G A G C T G C A T C G A T A T G C G T A C | 1e-90 | -2.081e+02 | 8.86% | 3.70% | 52.7bp (68.2bp) | ETS:E-box(ETS,bHLH)/HPC7-Scl-ChIP-Seq(GSE22178)/Homer(0.903) More Information \| Similar Motifs Found | motif file (matrix) |
| 14 | T A C G C G A T C T A G A C T G A T C G G T A C C T A G A T C G C T G A C T A G T G A C A G T C C T G A | 1e-90 | -2.077e+02 | 23.52% | 14.86% | 56.1bp (61.4bp) | Sp5(Zf)/mES-Sp5.Flag-ChIP-Seq(GSE72989)/Homer(0.938) More Information \| Similar Motifs Found | motif file (matrix) |
| 15 | G T A C C G T A C T A G A G T C C G A T A C T G G C T A G A T C C T G A | 1e-87 | -2.008e+02 | 43.07% | 32.44% | 56.5bp (64.8bp) | Ap4(bHLH)/AML-Tfap4-ChIP-Seq(GSE45738)/Homer(0.849) More Information \| Similar Motifs Found | motif file (matrix) |
| 16 | A T C G G A C T C A T G C T A G A G T C C G A T A C T G G A T C G C A T C T G A C T G A | 1e-86 | -1.988e+02 | 25.59% | 16.78% | 57.0bp (64.4bp) | ERF15(AP2EREBP)/colamp-ERF15-DAP-Seq(GSE60143)/Homer(0.803) More Information \| Similar Motifs Found | motif file (matrix) |
| 17 | A G T C A C G T A C T G G T A C G A C T C T A G C G T A | 1e-71 | -1.650e+02 | 43.81% | 34.12% | 57.4bp (63.5bp) | NRL/MA0842.1/Jaspar(0.787) More Information \| Similar Motifs Found | motif file (matrix) |
| 18 | C T A G G A T C C G A T C T A G G C A T A G T C C G T A A C T G | 1e-67 | -1.550e+02 | 33.74% | 25.02% | 56.7bp (62.3bp) | achi/MA0207.1/Jaspar(0.884) More Information \| Similar Motifs Found | motif file (matrix) |
| 19 | C A T G G T A C G C A T T A C G C G T A A C G T A C G T C G T A | 1e-61 | -1.413e+02 | 22.52% | 15.41% | 58.3bp (63.5bp) | POU6F2/MA0793.1/Jaspar(0.782) More Information \| Similar Motifs Found | motif file (matrix) |
| 20 | A G T C A G T C A T G C G T A C A G T C T C G A A G T C C T G A | 1e-54 | -1.259e+02 | 10.60% | 5.96% | 51.7bp (59.9bp) | Egr1(Zf)/K562-Egr1-ChIP-Seq(GSE32465)/Homer(0.805) More Information \| Similar Motifs Found | motif file (matrix) |
| 21 | A G T C A G T C A G T C A T G C A T G C G A T C A G T C A G T C A C G T | 1e-49 | -1.146e+02 | 12.79% | 7.87% | 56.6bp (61.6bp) | Maz(Zf)/HepG2-Maz-ChIP-Seq(GSE31477)/Homer(0.891) More Information \| Similar Motifs Found | motif file (matrix) |
| 22 | A G C T A C G T A C G T A G T C A G C T C T G A C T G A A C T G C G T A C G T A | 1e-47 | -1.095e+02 | 5.10% | 2.22% | 54.0bp (65.0bp) | STAT5(Stat)/mCD4+-Stat5-ChIP-Seq(GSE12346)/Homer(0.967) More Information \| Similar Motifs Found | motif file (matrix) |
| 23 | A C G T A C G T A C G T A C T G C G T A C G T A A T C G | 1e-45 | -1.045e+02 | 9.12% | 5.18% | 53.8bp (63.4bp) | CHR(?)/Hela-CellCycle-Expression/Homer(0.805) More Information \| Similar Motifs Found | motif file (matrix) |
| 24 | C A G T C T G A C T G A C T G A T G A C G C T A C T A G T C A G C T G A C T G A A T G C C G A T A C T G | 1e-44 | -1.029e+02 | 1.45% | 0.22% | 56.1bp (60.5bp) | SpiB(ETS)/OCILY3-SPIB-ChIP-Seq(GSE56857)/Homer(0.777) More Information \| Similar Motifs Found | motif file (matrix) |
| 25 | T G C A T C G A G T A C G A T C C T A G A G T C G C A T T C G A T C G A T G A C T C A G | 1e-44 | -1.023e+02 | 3.63% | 1.35% | 54.2bp (67.8bp) | RUNX3/MA0684.1/Jaspar(0.778) More Information \| Similar Motifs Found | motif file (matrix) |
| 26 | A C G T A T C G T C G A G A T C C A T G A G C T T C G A C G T A A G T C | 1e-44 | -1.020e+02 | 7.13% | 3.73% | 56.8bp (62.4bp) | CST6/MA0286.1/Jaspar(0.882) More Information \| Similar Motifs Found | motif file (matrix) |
| 27 | A G C T T A C G C T A G A G C T C G T A C T G A G A T C T A C G | 1e-41 | -9.575e+01 | 10.63% | 6.51% | 55.9bp (61.8bp) | MYB118(MYB)/colamp-MYB118-DAP-Seq(GSE60143)/Homer(0.830) More Information \| Similar Motifs Found | motif file (matrix) |
| 28 | C T G A C A T G G A T C A C T G T A C G C G A T C T G A A T C G A C G T | 1e-40 | -9.342e+01 | 4.23% | 1.81% | 58.9bp (61.8bp) | ETV6/MA0645.1/Jaspar(0.714) More Information \| Similar Motifs Found | motif file (matrix) |
| 29 | C G T A C T G A G T A C A C G T A C T G G T A C G T A C C G T A | 1e-40 | -9.300e+01 | 10.77% | 6.67% | 56.6bp (66.1bp) | Myb/MA0100.2/Jaspar(0.960) More Information \| Similar Motifs Found | motif file (matrix) |
| 30 | G T C A C A G T A T G C C G A T A T C G G C A T A G C T G T C A C G A T G A T C C G T A A T C G G A T C | 1e-37 | -8.730e+01 | 10.70% | 6.74% | 55.1bp (63.5bp) | Unknown5/Drosophila-Promoters/Homer(0.748) More Information \| Similar Motifs Found | motif file (matrix) |
| 31 | T A G C T C G A C T G A T G A C A G T C C A T G G T A C G T C A C A T G C G A T T C G A | 1e-37 | -8.677e+01 | 2.32% | 0.71% | 52.4bp (64.6bp) | Bgb::run/MA0242.1/Jaspar(0.763) More Information \| Similar Motifs Found | motif file (matrix) |
| 32 | A T C G A T G C C T G A A T G C G A C T G A C T G A T C G T A C T G A C A G T C G A C T | 1e-37 | -8.637e+01 | 2.65% | 0.90% | 53.9bp (59.2bp) | RO3G\_00049(RRM)/Rhizopus\_oryzae-RNCMPT00205-PBM/HughesRNA(0.853) More Information \| Similar Motifs Found | motif file (matrix) |
| 33 | A T C G T A G C G C T A C T A G T A C G T A G C C A G T A T C G C A G T A C T G C T A G A C T G | 1e-36 | -8.515e+01 | 6.98% | 3.87% | 55.5bp (60.8bp) | ETS:RUNX(ETS,Runt)/Jurkat-RUNX1-ChIP-Seq(GSE17954)/Homer(0.730) More Information \| Similar Motifs Found | motif file (matrix) |
| 34 | C T G A A T G C G T A C A G T C A G C T A G C T C G A T A T C G G C T A C G A T A T G C G C A T A C G T | 1e-31 | -7.359e+01 | 1.00% | 0.14% | 44.6bp (49.2bp) | pan/dmmpmm(Bergman)/fly(0.947) More Information \| Similar Motifs Found | motif file (matrix) |
| 35 | T C A G G A T C G T A C C T G A T A G C A G T C C G T A A C T G C T G A C A T G T C A G C T A G G T A C C T A G | 1e-31 | -7.162e+01 | 1.74% | 0.50% | 59.6bp (62.0bp) | BORIS(Zf)/K562-CTCFL-ChIP-Seq(GSE32465)/Homer(0.916) More Information \| Similar Motifs Found | motif file (matrix) |
| 36 | A T G C C T G A G A C T G C A T G T C A T C G A G A C T A G T C C T G A | 1e-28 | -6.521e+01 | 2.63% | 1.06% | 57.6bp (57.4bp) | Pdx1(Homeobox)/Islet-Pdx1-ChIP-Seq(SRA008281)/Homer(0.867) More Information \| Similar Motifs Found | motif file (matrix) |
| 37 | A T G C G A T C C G A T C G T A C G A T A G T C C T A G | 1e-27 | -6.379e+01 | 12.41% | 8.71% | 55.9bp (63.4bp) | DAL80/MA0289.1/Jaspar(0.827) More Information \| Similar Motifs Found | motif file (matrix) |
| 38 | C G T A C T A G T A G C G C T A T A C G T G C A T A G C C G T A C T A G G T A C C G A T A C T G | 1e-27 | -6.374e+01 | 6.00% | 3.48% | 55.3bp (61.0bp) | Myog/MA0500.1/Jaspar(0.772) More Information \| Similar Motifs Found | motif file (matrix) |
| 39 | C T A G A G C T A G C T A C G T G A T C A G T C G C T A A C G T T C A G C T A G G A T C C T G A T C G A A G T C | 1e-26 | -6.182e+01 | 1.72% | 0.54% | 53.2bp (56.3bp) | Rfx1/MA0509.1/Jaspar(0.954) More Information \| Similar Motifs Found | motif file (matrix) |
| 40 | G A C T C T A G G C T A A T G C A C T G C G A T A G C T | 1e-26 | -6.109e+01 | 7.62% | 4.81% | 54.9bp (64.8bp) | SKO1/Literature(Harbison)/Yeast(0.918) More Information \| Similar Motifs Found | motif file (matrix) |
| 41 | C G T A C T A G G T C A C A G T C G T A T G C A C T G A T A C G G T C A A C G T A G T C G A C T T A G C | 1e-26 | -6.022e+01 | 1.86% | 0.64% | 59.5bp (57.4bp) | GATA2/MA0036.2/Jaspar(0.753) More Information \| Similar Motifs Found | motif file (matrix) |
| 42 | C A T G A C T G T G C A T C G A T C G A A G T C A G T C C G T A T A G C G T C A T A G C C G A T A C T G A G T C | 1e-26 | -5.998e+01 | 0.58% | 0.04% | 54.4bp (45.4bp) | RUNX(Runt)/HPC7-Runx1-ChIP-Seq(GSE22178)/Homer(0.705) More Information \| Similar Motifs Found | motif file (matrix) |
| 43 | T G C A T A G C G A C T G T C A G T A C T C G A T G C A G A C T G C A T G A T C T G A C G A T C | 1e-25 | -5.805e+01 | 1.58% | 0.49% | 58.3bp (63.3bp) | GFY(?)/Promoter/Homer(0.992) More Information \| Similar Motifs Found | motif file (matrix) |
| 44 | C A G T C T A G T C G A C T G A A C T G G T C A T C G A T C G A G T A C G T A C A C T G G T A C G T C A C T A G | 1e-23 | -5.487e+01 | 0.98% | 0.21% | 49.4bp (54.0bp) | Bgb::run/MA0242.1/Jaspar(0.726) More Information \| Similar Motifs Found | motif file (matrix) |
| 45 | A G T C T C G A A G T C A C G T A G T C A T G C A G T C A G T C | 1e-23 | -5.377e+01 | 18.86% | 14.72% | 57.5bp (64.1bp) | KLF16/MA0741.1/Jaspar(0.858) More Information \| Similar Motifs Found | motif file (matrix) |
| 46 | A G T C T G A C G T A C A G T C G A T C A C G T T C A G T G A C G A C T C T A G | 1e-21 | -4.925e+01 | 3.65% | 1.95% | 55.7bp (60.1bp) | Unknown-ESC-element(?)/mES-Nanog-ChIP-Seq(GSE11724)/Homer(0.771) More Information \| Similar Motifs Found | motif file (matrix) |
| 47 | T C A G A C T G C G T A G A T C G A C T A C G T A G T C A G T C A G C T T C A G G C A T A C T G | 1e-21 | -4.880e+01 | 0.82% | 0.16% | 54.8bp (62.0bp) | Ets1-distal(ETS)/CD4+-PolII-ChIP-Seq(Barski\_et\_al.)/Homer(0.793) More Information \| Similar Motifs Found | motif file (matrix) |
| 48 | C G A T C T A G T A G C C T A G T A G C T G C A C A G T A T C G A G T C A C T G A G T C | 1e-20 | -4.725e+01 | 2.55% | 1.20% | 59.4bp (62.8bp) | NRF(NRF)/Promoter/Homer(0.977) More Information \| Similar Motifs Found | motif file (matrix) |
| 49 | A G C T A C T G G C T A A C G T C A G T C A T G T G C A A C G T T A C G C G A T A T C G C A T G A C T G T C G A | 1e-19 | -4.594e+01 | 1.04% | 0.28% | 53.5bp (56.5bp) | PBX1/MA0070.1/Jaspar(0.683) More Information \| Similar Motifs Found | motif file (matrix) |
| 50 | G T A C A G C T A T G C T A C G A G C T A G T C G T C A G A T C C T A G A C G T A T C G T G C A | 1e-19 | -4.444e+01 | 0.87% | 0.20% | 49.0bp (54.9bp) | E-box(bHLH)/Promoter/Homer(0.939) More Information \| Similar Motifs Found | motif file (matrix) |
| 51 | T C A G C A G T A C G T G T A C C G A T A G T C A C T G A G T C A C T G T C G A | 1e-19 | -4.376e+01 | 0.84% | 0.19% | 56.4bp (78.4bp) | GFX(?)/Promoter/Homer(0.871) More Information \| Similar Motifs Found | motif file (matrix) |
| 52 | A T G C A C G T A C T G C T G A A C G T C G T A C G T A C A T G C G T A G C T A C G A T A G T C C G T A C G A T | 1e-18 | -4.208e+01 | 0.34% | 0.02% | 52.3bp (51.9bp) | elt-3/MA0542.1/Jaspar(0.789) More Information \| Similar Motifs Found | motif file (matrix) |
| 53 | T C G A T G C A C T G A A C T G T A G C G T A C G A C T G A C T C G A T A T C G G T C A C G A T | 1e-17 | -4.125e+01 | 1.97% | 0.87% | 56.6bp (58.3bp) | PB0040.1\_Lef1\_1/Jaspar(0.739) More Information \| Similar Motifs Found | motif file (matrix) |
| 54 | A G C T G T C A A C T G C G A T C A T G A C T G A C G T C T G A G C T A T G A C | 1e-14 | -3.395e+01 | 0.77% | 0.21% | 56.4bp (61.7bp) | RFX1/MA0365.1/Jaspar(0.697) More Information \| Similar Motifs Found | motif file (matrix) |
| 55 | G T C A C T A G C G A T G A T C C T G A T A G C A T C G | 1e-14 | -3.294e+01 | 1.80% | 0.86% | 58.2bp (64.0bp) | Pax2/MA0067.1/Jaspar(0.814) More Information \| Similar Motifs Found | motif file (matrix) |
| 56 | G C A T A C T G A C G T G C A T C G T A C A G T G A T C G A C T G C T A G C A T G T A C C G T A C G T A | 1e-13 | -3.080e+01 | 0.66% | 0.17% | 59.2bp (55.9bp) | PB0021.1\_Gata3\_1/Jaspar(0.750) More Information \| Similar Motifs Found | motif file (matrix) |
| 57 | C G T A A C T G T C G A A G C T G T C A A G T C C G A T C T G A A G C T A G T C A C G T G C T A A T G C C G A T | 1e-12 | -2.850e+01 | 0.22% | 0.01% | 61.1bp (45.7bp) | GATA(Zf),IR3/iTreg-Gata3-ChIP-Seq(GSE20898)/Homer(0.736) More Information \| Similar Motifs Found | motif file (matrix) |
